# Hardship financing of healthcare among rural poor in Orissa, India

**DOI:** 10.1186/1472-6963-12-23

**Published:** 2012-01-27

**Authors:** Erika Binnendijk, Ruth Koren, David M Dror

**Affiliations:** 1 Institute of Health Policy and Management, Erasmus University Rotterdam, P.O. Box 1738, 3000 DR Rotterdam, The Netherlands; 2 Felsenstein Medical Research Center, Tel Aviv University Sackler Faculty of Medicine, Ramat Aviv, Tel Aviv, Israel; 3 Micro Insurance Academy, 246 Sant Nagar, East of Kailash, New Delhi 110065, India

## Abstract

**Background:**

This study examines health-related "hardship financing" in order to get better insights on how poor households finance their out-of-pocket healthcare costs. We define hardship financing as having to borrow money with interest or to sell assets to pay out-of-pocket healthcare costs.

**Methods:**

Using survey data of 5,383 low-income households in Orissa, one of the poorest states of India, we investigate factors influencing the risk of hardship financing with the use of a logistic regression.

**Results:**

Overall, about 25% of the households (that had any healthcare cost) reported hardship financing during the year preceding the survey. Among households that experienced a hospitalization, this percentage was nearly 40%, but even among households with outpatient or maternity-related care around 25% experienced hardship financing.

Hardship financing is explained not merely by the wealth of the household (measured by assets) or how much is spent out-of-pocket on healthcare costs, but also by when the payment occurs, its frequency and its duration (e.g. more severe in cases of chronic illnesses). The location where a household resides remains a major predictor of the likelihood to have hardship financing despite all other household features included in the model.

**Conclusions:**

Rural poor households are subjected to considerable and protracted financial hardship due to the indirect and longer-term deleterious effects of how they cope with out-of-pocket healthcare costs. The social network that households can access influences exposure to hardship financing. Our findings point to the need to develop a policy solution that would limit that exposure both in quantum and in time. We therefore conclude that policy interventions aiming to ensure health-related financial protection would have to demonstrate that they have reduced the frequency and the volume of hardship financing.

## 1. Background

While we know that the biggest part of health expenditures in India is paid by health-seekers themselves when getting care, we know much less about how those costs are met. Evidence confirms that out-of-pocket spending on healthcare absorb more than one quarter of household resources net of food costs in at least one-tenth of all households in India [1]. All over India, the level of out-of-pocket spending is 69.5% of total health expenditures [2]. This considerable burden warrants a better understanding of how poor households finance these out-of-pocket healthcare costs. This article focuses on this very question, using data from Orissa, where out-of-pocket spending represents nearly 80% of health expenditure [2].

The literature dealing with financing of out-of-pocket healthcare cost includes definitions of "catastrophic" healthcare expenditures when spending exceeds an essentially arbitrary threshold. Xu et al. [3] fix the threshold at 40% of disposable income net of subsistence needs; Russell [4] and Van Doorslaer et al. [1] use a threshold of 10% of total annual household income. However, these methods fail to recognize that a uniform threshold might represent varying levels of hardship. For example, spending 10% by a poor household could mean withdrawing a child from school or skipping a meal, while the same spending level would not entail any immediate consequence for a richer household [5,6]. Also the timing of the payment could cause different cash-flow problems. As most rural poor households in India have irregular flows of income, they would find it easier to pay during harvest season (when they have income from selling the crop or from work as agricultural labourers) than in other times of the year. Morduch and Rutherford [7] reported this cash-flow pattern to hold true in most low-income countries.

We therefore assess the hardship a household faces as a consequence of health expenses not merely by the amount spent, but by the additional costs to the direct cost of healthcare related to how the out-of-pocket spending is financed. This is in line with notions put forward by Flores et al. [8] and Kruk et al. [5].

Three sources of financing out-of-pocket healthcare costs can be distinguished: paying from current income or savings; borrowing with zero interest (e.g. from family and friends), and borrowing with interest or selling assets. The first two categories may be regarded as less burdensome than the third [5,9,10], because selling assets or borrowing money with interest usually entails a cost. This cost is self-explanatory in the case of interest on loans [11]. Selling assets also generates costs, such as losses when assets are sold at less than optimal price, or future income loss due to the sale of income-generating assets (like land or livestock). Thus, we say that households incur "hardship financing" when they are exposed to a less stable or worsened financial state brought about by additional costs/losses due to borrowing or selling assets. This definition follows Kruk et al. [5], with the notable modification that we only consider borrowing with interest instead of any borrowing. Our adjustment is in agreement with previous findings [12] and with our investigations that confirmed that borrowing from family/friends for healthcare purposes is indeed mostly interest-free.

Many of the previous publications regarding the financing sources for out-of-pocket payments for healthcare in developing countries, and in India, are related to financing of care for specific diseases [4,13-19] and mostly based on small-scale surveys. Some studies look at specific population segments [9,20], others used data available on a whole country level [5,8,10,21]. Kruk et al. [5], whom we follow in the definition of "hardship financing", looked at nationally representative household surveys of 40 low- and middle-income countries, whereas we look at rural (and largely tribal) poor in three districts of Orissa, one of the poorest states in India. By focusing on this target population, we examine hardship financing of out-of-pocket payments for healthcare expenditures among the most vulnerable segment of society in India. Our goal is to identify the parameters affecting households' risk to resort to hardship financing.

## 2. Methods

### 2.1. Setting and Sampling

We used data from a household survey undertaken early in 2009 in the rural areas of Kalahandi, Khorda, and Malkangiri districts of the state of Orissa. Orissa, the eleventh largest state of India by population (41,947,358) with 83% of population being rural [22,23], is located on the Bay of Bengal at the east coast of India. The average monthly per capita consumer expenditure (MPCE) of INR459 (PPP$30.6) for rural Orissa, is the lowest of all states [24]. The household survey questionnaire was translated into Oriya (the local language), back translated for verification, and pre-tested among 80 households in the area. Surveyors who spoke local dialects fluently conducted the survey.

We followed a three-stage sampling procedure. (i) The sites in Orissa were selected due to a relationship with 11 NGOs^1 ^that invited the research team to conduct a baseline study (prior to launching a development project among their members). (ii) Within each district, villages were selected randomly from among those selected by the NGOs for the development project (27 villages in Kalahandi, 22 in Khorda, 31 in Malkangiri). (iii) Stage three entailed random sampling of two equal sub-cohorts in each village: 'member households' and 'non-member households' (comparator group). Member households (i.e. households that included at least one person who was a member of a Self-Help Group (SHG^2^) linked to one of the respective NGOs) were selected randomly out of the membership list. Non-member households were sampled randomly with the use of line sampling (from the centre of the village 4 lines were drawn in the four winds directions, "the four winds technique") [25]. We interviewed a total of 5,383 households representing 25,606 individuals (with a similar number of member and non-member households in every village, totalling 2,688 member and 2,695 non-member households and with a similar number of households from each district, totalling 1820 households from Kalahandi, 1763 households from Khorda and 1800 households from Malkangiri). 100% of the sample interviewed was rural.

At the time of the rollout of the survey there was no local ethics committee in place in Orissa, India. We however held a two-day workshop in preparation of the study in which we discussed the ethical aspects of the study with scholars and senior scholars from India. Informed consent of the respondents was obtained at the beginning of the interviews and we kept participants' names confidential in data recording and analysis.

### 2.2. Data

The household survey questionnaire included questions on socioeconomic status: education of household head, occupation of household head, source of drinking water, toilet facility and caste. Under the Constitution of India, the government has "scheduled" certain backward Indian classes or groups [hence Scheduled Castes [26] or Scheduled Tribes [27], and "Other backward castes"], with the view to promoting their welfare. Scheduled Tribes (Tribals or Adivasis) are mostly not Hindu and thus out of the caste system and are considered the most disadvantaged economically. Scheduled Castes (Dalits and those sometimes labelled "Untouchable") are considered at the bottom of caste hierarchy. The list of Other Backward Castes is quite dynamic and changes from time to time in many states. All other castes are described here as General Caste.

For household income, we followed the method adopted by the Indian National Sample Survey Organization to obtain a proxy (monthly per capita consumer expenditure) through questions on many items of household expenditure (expenditures on food, clothing, fuel, etc.) [24]. In our study, unlike the National Sample Survey Organization, we did not include health expenditure, because we seek to identify patterns of financing of healthcare [6,8]. We label this proxy for socioeconomic status as "income-proxy".

We also developed an asset-index as proxy for socioeconomic status by performing a principal component analysis (PCA) on various aspects of household assets, following the guidelines of Vyas and Kumaranayake [28]. PCA is a statistical technique used for data reduction. We included the following variables: house type, source of lighting, way of cooking, land ownership, various consumer durables (radio, motor cycle, telephone, etc.), possession of animals (cattle, sheep, chickens, etc.). Size of land and possession of animals were included as continuous variables, the other variables as binary (yes/no) variables. We then used the factor scores of each of the variables from the first principal component as weights and computed a total for each household: the household's socioeconomic score. Characteristic of this score is that it has a mean equal to zero and a standard deviation equal to one; the higher the score, the higher the implied socioeconomic status or wealth of that household. As we deal in this paper with health-related issues, source of drinking water and toilet facility were not included in the PCA given their possible direct relation with health status [28,29].

Besides indicators of socioeconomic status, the household survey questionnaire included questions on healthcare utilization and cost. The households were asked whether they had incurred expenditures for outpatient care, hospital admittances and maternity-related care in the year preceding the survey. Hospital admittances reflected cases with inpatient stay exceeding 24 hours. Stays in hospital of less than 24 hours were counted under outpatient care, together with consultations with a healthcare practitioner, and payment for medicines or tests in an outpatient setting. Maternity-related utilization included delivery and pre- and post-natal care. Respondents were asked to estimate total direct medical expenditures of the household in the year preceding the survey, as well as the expenditures for hospital admittances in the household in the year preceding the survey. More detailed information on outpatient care utilization and cost was queried for one month preceding the survey. Chronic illness in the household was identified by a set of questions related to symptoms, length of illness and regular medicine use.

We asked households also what hospital they would go to in case of a hospitalization of more than 24 hours, what kind of hospital this is, and the distance (travel time in minutes) to this hospital. Similar questions were asked related to the practitioner for outpatient care.

Households that reported healthcare costs for hospitalizations, outpatient care, or maternity-related care during the year preceding the survey were asked how they financed each type of these cost. Sources of financing included using current income, money received as gift, savings, money obtained from selling assets, money obtained through borrowing (borrowing options included relatives, friends/neighbours, bank, moneylender, or microfinance e.g. local microfinance institution or SHG), health insurance, and other sources. Households could report as many sources of financing as relevant. With respect to borrowing we also queried how much was borrowed from each of these sources to pay for healthcare costs. In this paper we ignore three sources since they turned out to be negligible (e.g. 1.4% of households with any health expenditure reported gifts, 0.02% reported health insurance and 0.7% reported "other").

We categorized a combination of financing sources as hardship financing: selling assets or borrowing money with interest from bank, microfinance or moneylender. If a household had reported using at least one of these financing sources, this household was categorized as having had hardship financing. Households that reported using only current income and/or savings and/or borrowing from relatives or friends/neighbours were defined as having had no hardship financing.

### 2.3. Analysis

We investigated factors influencing the risk of households to need hardship financing when paying for healthcare costs with the use of a multivariate analysis. We applied a logistic regression (logit model) as the outcome variable is a binary variable (yes/no hardship financing). Only households that had healthcare costs in the year preceding the survey were included in the regression. The explanatory variables were included in the model in a stepwise inclusion procedure

Data is analyzed using STATA version 11. The unit of analysis is the household, reflecting the fact that in rural low-income countries, many decisions on paying for healthcare are taken at that level rather than by individuals [30].

Statistical significance of difference has been shown at levels of 10%, 5% and 0.1% throughout the paper. When ANOVA is used we show significance with *, ** and *** respectively; when Pearson Chi-square is used we show significance with^†^, ^†† ^and^††† ^respectively. In case of the logistic regression statistical significance of the coefficient (Z-test) is shown at levels of 5%, 1% and 0.1% with *, ** and *** respectively.

All amounts, reported in Indian Rupee (INR) during the survey, were converted into international dollars (Purchasing Power Parity, PPP$) using the exchange rate of PPP$1 = INR 16.389 for 2009 [31].

## 3. Results

### 3.1. Socioeconomic profile

The socioeconomic profile of the sampled population is summarized in Table 1. The majority of the studied population is from Scheduled Caste or Tribe. The majority of the household heads have no or very little education and work as daily wage labourers or are self-employed in agriculture. The income of our sampled population in Orissa was on average below the extreme poverty anchor of PPP$1.08 per person and day (defined by the World Bank in 1993, equalling PPP$1.71 p.p.p.d. when adjusted to the survey year 2009 and to India) [32,33]. Most households have no toilet and get drinking water from a shared tap in the village or hand pump/well. The households consist on average of 4.8 persons of which 8% are infants (0-4 years old) and 8% are elderly (60 years and older).

**Table 1:** Demographics & socioeconomic status

	Mean (± SE)^a^
Income-proxy p.p.p.m (PPP$)^b^	32.41 (± 0.29)*****

Asset-index^c^	0.00 (± 0.03)*****

Household size	4.76 (± 0.02)*****

Ratio infants (0-4) in household	0.082 (± 0.002)****

Ratio elderly (60 and older) in household	0.075 (± 0.002)****

	% of total

Caste^d^	
Scheduled Tribe	30.0
Scheduled Caste	22.3
Other Backward Caste	31.3
General Caste	16.5

Education level household head	
No education	51.4
Class 1-5	22.2
Class 6-10	23.8
Class 11 and higher	2.7

Occupation household head	
Self-employed agriculture	38.7
Self-employed business/trade	16.1
Regular Salaried employee	4.6
Daily wage labourer	30.8
Not working	9.9

Source of drinking water	
Own tap	9.9
Shared tap	53.9
Hand-pump/well	36.2

Toilet facility	
Own flush toilet	4.4
Own pit toilet	4.9
Shared toilet	1.0
No toilet	89.7

^a ^SE = Standard Error.

^b ^Income is proxied as monthly per capita consumer expenditure through questions on many items of household expenditure and expressed in Purchasing Power Parity International Dollar.

^c ^Asset-index is a proxy for socioeconomic status based on various aspects of household assets. The index is calculated using a principal component analysis (PCA).

^d ^Caste is a proxy for socioeconomic status in India. Scheduled Castes (Dalits and those sometimes labelled "untouchable") are considered at the bottom of caste hierarchy. The list of Other Backward Castes is quite dynamic and changes from time to time in many states. All other castes are described here as General Caste.

The socioeconomic status indicators from the National Sample Survey Organization for rural Orissa show similar patterns as our aggregated total population (table 1): income-proxy PPP$ 28.9 p.p.p.m., household size 4.6 persons) [24].

### 3.2. Morbidity, healthcare availability, utilization and cost

Information on morbidity, availability of healthcare, utilization of healthcare and healthcare expenditures of the sampled population are summarized in Table 2.

**Table 2:** Morbidity, healthcare availability, utilization and cost in the sampled population

	Mean (± SE)^a^
Total health expenditure last year for household (PPP$)^b^	167.34 (± 5.14)

Distance to preferred hospital (in minutes)	52.48 (± 0.54)

Distance to preferred primary care practitioner (in minutes)	30.40 (± 0.40)

	% of total

Household with chronic ill person	10.5

Household with hospitalization costs last year	23.5

Household with outpatient care costs last year	83.8

Household with maternity costs last year	14.4

Household with any healthcare costs last year	85.1

Hospital household usually goes to	

Private	6.3

Public	93.7

Preferred primary care practitioner household usually goes to	

Traditional healer	36.6

Government facility	50.4

Unqualified private doctor (non-MBBS)^c^	7.6

AYUSH practitioner^d^	3.2

Qualified private doctor/specialist (MBBS)	2.2

^a ^SE = Standard Error.

^b ^Total health expenditure last year for household expressed in Purchasing Power Parity International Dollar.

^c ^Unqualified private doctor (non-MBBS) is a doctor practicing allopathic medicine without having a medical degree (Medical Bachelor and Bachelor of Surgery).

^d ^AYUSH is the aggregate of all qualified systems of traditional medicines in India: Ayurveda, Yoga and Naturopathy, Unani, Siddha and Homeopathy.

Around 85% of the sampled households had health expenditures in the year preceding the survey, and almost 24% of households had to meet hospitalization costs in the same period. Around 10% of the sampled households have a chronically ill person in the household.

Average health expenditures represented about 9% of the average income-proxy for the sampled population (income-proxy p.p.p.m. and household size table 1 total health expenditure for the household last year table 2).

Households usually go to a public facility for hospitalizations of more than 24 hours. In about half of the cases they go to a public facility for outpatient care. To go to the facility where they would go to for a hospitalization takes a little bit more than 50 minutes travel time; the facility for outpatient care is on average 30 minutes away from their homes.

In Additional Files 1 and 2 the same information of Table 1 and 2 is shown separate for the member and non-member sub-cohorts (defined in the methods section). The difference between SHG members and non-members in morbidity, healthcare utilization and cost is not significant for most indicators. The difference in socioeconomic profile between the member and non-member cohorts seems to be significant, pointing to a higher socioeconomic status for SHG members. However, for the indicators that are significantly different, this difference is rather small and probably immaterial. Therefore we aggregated the two sub-cohorts for the descriptive statistics, but included the membership variable in the regression analysis.

### 3.3. Healthcare financing

**Figure 1:**
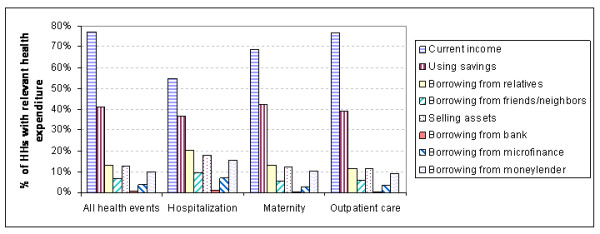
**Healthcare financing**.

We asked respondents how they financed their healthcare costs (Figure 1). The majority of the households reported to have used at least (some) of their current income and savings to pay for their health expenditures. However, the multiplicity of sources shows that households were often unable to fund all their health expenditures from their current income and savings alone.

**Figure 2:**
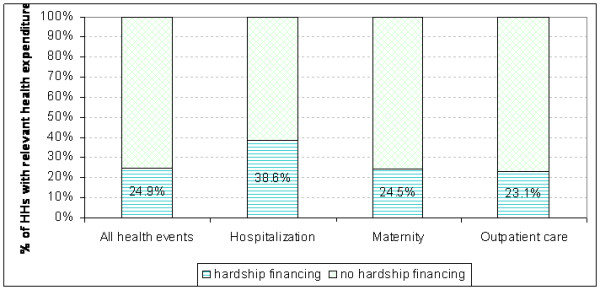
**Hardship financing**.

Households selling assets or borrowing money with interest in order to finance their healthcare were defined as households with hardship financing. Households that were able to finance their healthcare costs solely from current income, savings and/or borrowing without interest (from relatives or friends/neighbours) are defined as households with no hardship financing. Figure 2 shows the shares of households paying healthcare costs with hardship financing versus households that did not have to use hardship financing.

Overall, about 25% of the households (with any healthcare cost) had hardship financing during the year preceding the survey. Among households that experienced a hospitalization this percentage is much higher, nearly 40% had hardship financing. Quite unexpectedly, 23% of household that incurred outpatient costs also reported that they had to use hardship financing sources to pay for this outpatient care, as well as 25% of the households that had a maternity case in the reference period.

### 3.4. Parameters influencing the risk of hardship financing

We explored with the use of a logistic regression the factors that could influence the risk of households to need hardship financing when paying for healthcare costs (Table 3).

**Table 3:** Factors that influence the risk of hardship financing for households with healthcare costs (logistic regression)

	Coefficient	95% Confidence interval	
*Socioeconomic and demographic parameters*			
Log income-proxy^a^	0.1197	-0.0694	0.3089

Asset-index^b^	-0.1176***	-0.1698	-0.0655

Employment household head			
Salaried employee	*Reference*		
Self-employed in agriculture	0.8420**	0.3307	1.3533
Self-employed in business/trade	0.7521**	0.2176	1.2866
Daily wage labourer	0.9204**	0.4005	1.4404
Not working	0.7824**	0.2145	1.3503

Education household head			
No education	*Reference*		
Class 1-5	-0.0831	-0.2991	0.1329
Class 6-10	-0.1719	-0.3930	0.0492
Class 11 and higher	-0.0340	-0.6023	0.5343

Caste^c^			
Scheduled Caste	-0.1293	-0.4347	0.1761
Scheduled Tribe	0.2173	-0.0632	0.4978
Other Backward Caste	-0.1570	-0.4255	0.1116
General Caste	*Reference*		

Household size	0.0370***	-0.0135	0.0876

Ratio infants (0-4) in household	0.2015	-0.4490	0.8520

Ratio elderly (60+) in household	0.1851	-0.3583	0.7284

Source of water			
Own tap	*Reference*		
Shared tap	0.4873**	0.1733	0.8013
Hand pump/Well	0.3540*	0.0286	0.6794

Type of toilet			
Own flush toilet	*Reference*		
Own pit toilet	0.0556	-0.5192	0.6305
Shared toilet	0.7037	-0.2623	1.6697
No toilet	-0.0607	-0.4942	0.3728

*Morbidity parameters and health expenditures*			
Log health expenditures last year	0.7232***	0.6365	0.8099

Chronic illness in household			
No chronic ill	*Reference*		
Chronic ill	0.3779**	0.1301	0.6257

Hospitalization in household last year			
No hospitalization	*Reference*		
Hospitalization	0.7225***	0.5335	0.9116

*SHG membership in household*			
No SHG member	*Reference*		
SHG member^d^	0.2184**	0.0536	0.3833

*Location of residence*			
Khorda district	*Reference*		
Kalahandi district	1.3776***	1.1376	1.6176
Malkangiri district	0.1739	-0.0947	0.4424

Constant	-9.5496***	-11.1226	-7.9766

N	4121		
Likelihood ratio test:			
LR chi2(27)	1025.76		
Prob > chi2	0.00		
Pearson goodness-of-fit test:			
Pearson chi2(4091)	3930.87		
Prob > chi2	0.96		

*** Significance of coefficient p < 0.05 (Z-test)

*** Significance of coefficient p < 0.01 (Z-test)

*** Significance of coefficient p < 0.001 (Z-test)

^a ^Income is proxied as monthly per capita consumer expenditure through questions on many items of household expenditure and expressed in Purchasing Power Parity International Dollar.

^b ^Asset-index is a proxy for socioeconomic status based on various aspects of household assets. The index is calculated using a principal component analysis (PCA).

^c ^Caste is a proxy for socioeconomic status in India. Scheduled Castes (Dalits and those sometimes labelled "untouchable") are considered at the bottom of caste hierarchy. The list of Other Backward Castes is quite dynamic and changes from time to time in many states. All other castes are described here as General Caste.

^d ^A household was defined as SHG member if at least one person in the household was a member of a Self-Help Group (SHG) linked to one of the related NGOs.

#### Socioeconomic and demographic parameters

We found that, whereas the logarithm of the income-proxy was not a significant predictor of hardship financing, the asset-index was a highly significant and negative predictor (i.e. households with a lower asset-index have a higher propensity to have hardship financing). The education of the household head did not influence the probability to have hardship financing while his occupation did; households where the household head is self-employed in agriculture, self-employed in business, a daily wage labourer, or does not work due to any reason all have a significant higher likelihood to have hardship financing than households where the household head is a regular salaried employee. Caste, household size, the ratio of infants or the ratio of elderly in the household did not have a significant impact on hardship financing.

The minority (10%) of households with a better source of water are less likely to have hardship financing. The type of toilet used was not a significant explanatory variable.

#### Morbidity parameters & health expenditures

The logarithm of the total health expenditures during the year preceding the survey is a significant and positive predictor of hardship financing. Interestingly, having a chronically ill person in the household, or having experienced one or more events of hospitalization in the year preceding the survey are both significant independent predictors even after the overall health expenditures has been taken into account in the regression analysis.

### 3.5. Impact of SHG membership on hardship financing

The logistic regression (table 3) revealed that households where someone is a member of an SHG have a higher propensity to have hardship financing. This we found somewhat unexpected as we did not observe a big difference between the member and non-member sub-cohorts in demographics, socioeconomic status, morbidity, healthcare utilization and costs. Also the direction of the influence is somewhat unexpected. When looking at the demographics, SHG member households have a slightly higher asset-index, have more household heads that are salaried employees and more often have their own tap than non-member households (i.e. less poor households). And, from the logistic regression (table 3) it can be seen that, as expected, less poor households are less likely to have hardship financing. But, when controlled for these variables in the regression itself, it seems that SHG members are more likely to have hardship financing.

When looking at the financing sources used by members and non-members, we found that SHG members rely significantly more often on microfinance as a source of borrowing than non-members when paying for all health expenditures, hospital expenditures, or outpatient expenditures (4.4% vs. 2.3%, p < .001; 8.5% vs. 4.7%, p < 0.01; 3.7% vs. 2.1%, p < .01 respectively, Chi2). Non-members on the other hand make more frequently use of their savings in order to pay for hospital expenditures than members (23.8% vs. 17.0%, p < .01, Chi2).

### 3.6. Impact of location on hardship financing

**Figure 3:**
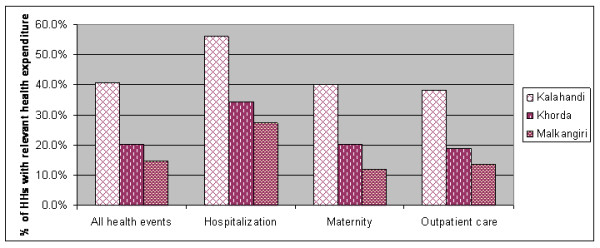
**Hardship financing in the three districts**.

When controlled for all features of individual households as variables included in the logistic regression, still the district in which a household resides remains a major predictor of the likelihood to have hardship financing (table 3). Therefore we explored this further. Figure 3 shows the difference in hardship financing between the districts.

By using a step-by-step inclusion procedure for the different variables in the regression, we found that the healthcare costs explained the difference in hardship financing between households in Malkangiri and Khorda districts. No other variable had this effect. The difference between Kalahandi on the one hand and Khorda and Malkangiri on the other hand remained unexplained with the current set of variables.

In the course of the examination we also checked other variables (e.g. preferred type of hospital, preferred practitioner for outpatient care, and distance (in minutes of travel time) to both preferred hospital and preferred outpatient practitioner) which were eliminated from the model as they did not proof to be statistically significant and weakened the overall model.

In order to find an explanation for the difference across the districts, we looked at alternative morbidity-related parameters. From our household survey we also have information on illness episodes and related costs for last month that cannot be annualized at the level of the single household. Therefore these parameters can serve to estimate morbidity of the district, but cannot be included in the regression analysis (Table 4).

**Table 4:** Additional explanatory variables across the three districts.

Parameter	Kalahandi	Khorda	Malkangiri
% of sampled household that reported illness last month	62.3%	61.6%	51.3%^††† d^

% of sampled household that had outpatient treatment last month	60.7%	59.8%	49.1%^††† d^

Outpatient cost of household that reported illness last month (± SE) (PPP$)^a^	56.65 (± 2.21)	71.74 (± 3.39)	34.78 (± 1.77) ***^e^

Income-proxy p.p.p.m. (± SE) (PPP$)^b^	29.94 (± 0.48)	36.16 (± 0.54)	31.35 (± 0.46) ***^b)^

Asset-index (± SE)^c^	-0.70 (± 0.04)	1.36 (± 0.06)	-0.62 (± 0.05) ***^f^

% of household with healthcare expenditures that reported borrowing from relatives and/or friends	15.6%	23.7%	14.2%^††† g^

*** Significance of overall difference across the three districts p< 0.001 (ANOVA)

^††† ^Significance of overall difference across the three districts p< 0.001 (Pearson Chi-square)

^a ^Average outpatient costs ± Standard Error, expressed in Purchasing Power Parity International Dollar.

^b ^Income is proxied as monthly per capita consumer expenditure through questions on many items of household expenditure and expressed in Purchasing Power Parity International Dollar.

^c ^Asset-index is a proxy for socioeconomic status based on various aspects of household assets. The index is calculated using a principal component analysis (PCA). SE = Standard Error

^d ^The difference between Kalahandi and Khorda is not significant for both indicators, p = .687 and p = .593 respectively Chi2

^e ^For each of the three districts the average cost is significantly different from any other district, p < .001 ANOVA

^f ^The difference in average asset-index between Kalahandi and Malkangiri is not significant (p = .163 ANOVA)

^g ^The difference in share between Kalahandi and Malkangiri is not significant, p = .282 Chi2

In both Kalahandi and Khorda around 62% of the households reported an illness last month and around 60% had outpatient treatment (in the form of consultation, medicines and/or tests) for that illness. In Malkangiri this percentage is lower: 51% of the households reported an illness last month and 49% had some form of outpatient treatment for that illness. The average cost of the outpatient treatment is the highest in Khorda (PPP$71.74), a bit lower in Kalahandi (PPP$56.65) and much lower in Malkangiri (PPP$34.78).

Although the parameters of the socioeconomic status of individual households were included in the regression, we wondered whether the average socioeconomic status of the district is associated with the risk of hardship financing. Therefore we also show the average socioeconomic status (income-proxy and asset-index) for each of the three districts in table 4. We found that the sampled population in Khorda is on average wealthier than the other two districts which are similar to each other.

Finally we examined whether the access of households to a social network of family and/or friends that can provide interest-free loans is different in the three districts. We checked this through the share of households with health expenditures that borrowed from family and/or friends (table 4). We found that households in Khorda had a better access to such a social network than households in Kalahandi and Malkangiri. It is interesting to note that a higher average asset-index is positively associated with higher interest-free borrowing from family/friends.

## 4. Discussion

In this study we found that many households experienced hardship financing. We defined hardship financing as being exposed to a less stable or worsened financial state brought about by additional costs/losses due to borrowing or selling assets. Based on the information in our household survey we cannot quantify losses due to selling assets. However, using the amounts borrowed from different sources as reported in our household survey and assuming a standard period of 12 months for all loans, an equal monthly repayment regime, and the following interest rates per annum (48% - moneylenders, 24% - microfinance, 12.5% - banks [12,34]), we can estimate the additional cost of the interest payment on the loans. Households that borrowed with interest from a bank, moneylender or microfinance paid, under the above assumptions, a mean interest amount of PPP$64. This adds almost 24% to their healthcare costs, and represents on average nearly 5% of overall annual household expenditure excluding health expenditure. Unlike common economic theory that borrowing or selling assets would have immediate welfare in the sense that the ill person could be treated, that welfare gain comes at a cost of welfare loss that extends over a much longer period of time. The added welfare comes at varying prices, which reflects not the value of the additional consumption (in this case the cost of care) but the varying cost of financing, which is more expensive for those that resort to moneylenders than those that can resort to cheaper sources of borrowing or do not have to borrow at all.

Hardship financing occurs not only when facing the high cost of inpatient care (nearly 40%) but also outpatient care (23%). This finding is in line with a previous study where it was found that the aggregated costs of outpatient care can exceed those of inpatient care among low-income households in India [35]. Interestingly our results reveal that maternity-related expenditures also cause hardship financing for a quarter of the households, although these events are known in advance and theoretically the household could save money and prepare for them. When considering the entire target population, the impact of hardship financing of outpatient care can be considered more severe than of inpatient care since 84% of the sampled households reported expenses due to outpatient care and only 24% had expenses due to inpatient care. Berman et al. [36] have reached a similar conclusion using a different methodology. These authors compared the number of households below poverty line before and after healthcare payment as a definition of impoverishment and found that outpatient care was more impoverishing than inpatient care for households in India.

Peters [37] reported that 40% of hospitalized patients (all-India average) had to sell assets or borrow money to pay for hospital costs. Duggal [38] found that among the poorest quintile in India, this percentage was 50%. One would expect our result for the rural poor communities of Orissa to be similar to the percentage found for the lowest quintile. The discrepancy however could be due to the difference in definition, as we considered as hardship financing only borrowing with interest. According to these and other results [5] it is reasonable to assume that poorer households have a higher risk to experience hardship financing. We wondered whether this difference still holds within our study-population, all of which is poor. We addressed this issue in our multivariate logistic regression by including two measures for wealth (income-proxy and asset-index). Interestingly we found that whereas a lower asset-index was associated with a higher risk of hardship financing, the association with income-proxy was not significant. This may well reflect the situation that in the informal economy many transactions are not monetized and the possession of assets is a more reliable indicator of socioeconomic status [39]. The negative association between asset-index and hardship financing could be due to asset-rich households for instance having a better chance of accessing social networks that would be more likely to give interest-free loans (non-hardship financing). It cannot be excluded though that the lower asset-index may be a result of, rather than the cause for, hardship financing, as we measure the asset-index after the health event.

Households where the household head is a salaried employee were least likely to need hardship financing compared to households where the head was self-employed in business or in agriculture, was a daily-wage labourer or did not work. As income was included in the regression as a separate parameter, the reason for the correlation with employment cannot be attributed solely to a difference in income. Perhaps the difference is due to having a steady rather than erratic flow of income. This finding would then be in agreement with our hypothesis that hardship financing can sometimes be caused by a time-gap between the inflow of income and outflow of health expenses.

From our logistic regression it turned out, as could be intuitively expected, that health expenditures in the last year were significantly associated with the risk for hardship financing. However, interestingly, having had a chronic illness or hospitalization in the household in the last year were also independent significant indicators for hardship financing. This means that the presence of chronic illness or hospitalization affected the risk of hardship financing in a way which was independent of the related expenses. Both chronic illness and hospitalization generate many indirect costs (loss of income of the chronic ill patient, loss of income of the hospitalized patient and/or caretaker and transportation costs) which could independently aggravate the situation leading to hardship financing. The recurring nature of outpatient expenditures related to a chronic illness could cause depletion of savings and attrition of goodwill of others to give interest-free loans and thus increase the need for hardship financing. In the case of hospitalizations one should note that poor households usually have to pay (some) costs upfront before admission to or treatment in hospital [38]. Therefore the unpredictable timing of hospital care and immediate need for large funds associated with such an event could increase the risk of hardship financing.

SHG membership surprisingly reported increased likelihood to have hardship financing, even though we have seen that SHG members are slightly better off than non-members (Additional File 1) and that better off households need less hardship financing. The explanation for this phenomenon might be that members have their savings tied up in the scheme and can therefore not liquefy those savings when needed. On the other hand, SHG members have an easier access to low-interest loans while non-members cannot easily access microfinance and low-interest loans. Therefore it is possible that SHG members would prefer this low-interest loan over borrowing from relatives/friends who may not be able to spare the money for a long time.

Finally it becomes very clear from the regression that the difference in hardship financing across the three districts could not be fully explained by the many household features included in the model. We found that the district where the household resides has a big significant independent effect on the risk of hardship financing. Using the step-wise regression method we found that when healthcare costs are introduced into the model, the difference in the risk of hardship financing between Malkangiri and Khorda became insignificant. The difference in hardship financing between Kalahandi on the one hand and Khorda and Malkangiri on the other hand remained unexplained with the current set of variables: living in Kalahandi could be associated with increased likelihood of hardship financing, because of higher utilization and costs of outpatient care compared to Malkangiri. In Khorda, both utilization and costs of outpatient care were similar to those observed in Kalahandi, yet the risk of hardship financing was significantly lower. This could be linked to the related finding that average income-proxy and asset-index in Khorda are higher than in Kalahandi (the asset index introduced in the regression takes into account the wealth of the individual household but the average asset index in the district reflects the wealth of the social network). This difference could suggest that households in Khorda have access to richer family/friends (better social network) that can provide more interest-free loans. This assumption gains credibility from the finding that a higher percentage of households in Khorda borrowed from relatives/friends, compared to Kalahandi (table 4).

While this study can play an important role in advancing the notion of hardship financing as a measure of the effectiveness of health financing policy alternative to the catastrophic spending method, there are some limitations to this study. Without adequate data one cannot conclude that the same findings would apply elsewhere. And, as the source of data is interviews with respondents, the regular limitations of self-reporting of incidence of illness and cost of care apply.

## 5. Conclusions

Our study sheds light on a hitherto understudied dimension of hardship of very poor rural groups occasioned by the need to raise funds to pay for healthcare out-of-pocket. We defined "hardship financing" as borrowing with interest or selling assets. The extra cost due to the interest payable on money borrowed with interest is far from negligible. We estimated the additional costs due to interest on loans to pay for illness-related costs at 24% of the health expenditure, a cost that represented nearly 5% of households' annual overall expenditure. The monetary value of the loss due to selling assets is also not zero, but hard to estimate. The hardship associated with these costs extends well beyond the duration of the health event.

This analysis has shown that hardship financing occurs not only in cases of expensive hospitalizations (40%) but also in many cases of expenditures for outpatient (23%) and maternity care (25%). Taking into account that the frequency of outpatient utilization is much higher, many more people actually face hardship financing due to outpatient care than due to inpatient care.

We have shown that possession of assets and having regular income-flow are predictors of lower expected hardship financing, and better predictors than the income-proxy of the household used in this study. The first parameter indicates the aggregate financial strength of the household in an environment where many economical transactions are not monetary. The second parameter is self-explanatory, as regular income makes it easier to plan future expenses based on stable future income. Interestingly, not only the assets of the households with out-of-pocket healthcare costs were negatively associated with hardship financing, but also the average wealth in the community in which the households resides. This indicates that hardship financing is also influenced by attributes of the social network the household can access; better access to a wealthier social network seems to increase the likelihood of obtaining interest-free loans.

Our study adds a qualitative dimension to understanding health-related financial exposure among rural poor households. Hardship financing is explained not only by how much is spent out-of-pocket on healthcare in nominal terms or relative to income or assets, but also by when the payment occurs, and, as is the case with chronic illness, its frequency and duration. This important finding that rural poor households are subjected to considerable and protracted financial hardship due to the indirect and longer-term deleterious effects of how they cope with out-of-pocket healthcare costs points to the need to develop a policy solution that would limit that exposure both in quantum and in time. We therefore conclude that policy interventions aiming to ensure health-related financial protection would have to demonstrate that they have reduced the frequency and the volume of hardship financing.

## 6. List of abbreviations

INR: Indian Rupee; MPCE: Monthly Per Capita Consumer Expenditures; NGO: Non-Government Organization; PCA: Principal Components Analysis; PPP$: Purchasing Power Parity International Dollar; p.p.p.m: per person per month; SE: Standard Error; SHG: Self-Help Group.

## Endnotes

1) The 11 grassroots NGOs linked with Madhyam Foundation, Bhubaneswar, Orissa included: (i) in Malkangiri: Parivartan, PUSPAC, SOMKS, SDS, ODC; (ii) in Kalahandi: Mahashakti Foundation, DAPTA, Lok Yojana, Sanginee; and (iii) in Khorda MVPS, DSS.

2) SHGs represent a unique approach to financial intermediation in communities. The approach combines access to low-cost financial services with a process of self-management and development for the SHG members. SHGs are seen to confer many benefits, both economic and social.

## 7. Competing interests

The authors declare that they have no competing interests.

## 8. Authors' contributions

DMD is the lead researcher and responsible for overall project management. EB, DMD and RK are responsible for the study concept and design. EB supervised the fieldwork and was responsible for the data management of the household survey. EB, RK and DMD (in order of contribution) analysed and interpreted the data and drafted the manuscript. All authors read, revised and approved the final version of the manuscript.

## 9. Authors' information

This work represents part of the requirements for the PhD thesis of EB at Erasmus University Rotterdam.

DMD, in addition to acting as principal investigator on this grant within his position as hon. Professor at Erasmus University Rotterdam, is also the Chairman of the Micro Insurance Academy.

## Pre-publication history

The pre-publication history for this paper can be accessed here:

http://www.biomedcentral.com/1472-6963/12/23/prepub

## Supplementary Material

Additional file 1 **Demographics & socioeconomic status disaggregated for members and non-members**. This file contains the same information of Table 1 on demographics and socioeconomic status but separate for the member and non-member sub-cohorts (as defined in the methods section). Click here for file

Additional file 2 **Morbidity, healthcare availability, utilization and cost disaggregated for members and non-members**. This file contains the same information of Table 2 on morbidity, healthcare availability, utilization and cost but separate for the member and non-member sub-cohorts (as defined in the methods section). Click here for file
