# Supplementary material for: Hardship financing of healthcare among rural poor in Orissa, India
Source: BMC Health Serv Res. 2012 Jan 27;12:23. doi: 10.1186/1472-6963-12-23 (PMC3317855; doi:10.1186/1472-6963-12-23)
Supplement: Additional file 1 — Demographics & socioeconomic status disaggregated for members and non-members. This file contains the same information of Table 1 on demographics and socioeconomic status but separate for the member and non-member sub-cohorts (as defined in the methods section). [file 1472-6963-12-23-S1.DOC]

Additional File 1

Demographics & socioeconomic status disaggregated for members and non-members

|  | Non-member sub-cohort^a^ | Member sub-cohort^a^ |
| --- | --- | --- |
|  | Mean (±SE)^b^ | Mean (±SE)^b^ |
| Income-proxy p.p.p.m (PPP$)^c^ | 31.98 (±0.42)***** | 32.84 (±0.39) *^NS^ |
| Asset-index^d^ | -0.16 (±0.04)***** | 0.16 (±0.05)***** |
| Household size | 4.66 (±0.04)***** | 4.86 (±0.03)***** |
| Ratio infants (0-4) in household | 0.085 (±0.003)**** | 0.078 (±0.002)**** |
| Ratio elderly (60 and older) in household | 0.080 (±0.003)**** | 0.070 (±0.003)**** |
|  |  |  |
|  | % of total | % of total |
| Caste^e^ |  |  |
| Scheduled Tribe | 30.8 | 29.2 |
| Scheduled Caste | 21.8 | 22.8 |
| Other Backward Caste | 29.8 | 32.7 |
| General Caste | 17.6 | 15.3 ^††^ |
| Education level household head |  |  |
| No education | 52.8 | 49.9 |
| Class 1-5 | 20.7 | 23.7 |
| Class 6-10 | 23.6 | 23.9 |
| Class 11 and higher | 02.9 | 02.5 ^††^ |
| Occupation household head |  |  |
| Self-employed agriculture | 38.5 | 38.9 |
| Self-employed business/trade | 14.6 | 17.6 |
| Regular Salaried employee | 03.9 | 05.3 |
| Daily wage labourer | 33.0 | 28.5 |
| Not working | 10.0 | 09.7 ^†††^ |
| Source of drinking water |  |  |
| Own tap | 08.7 | 11.0 |
| Shared tap | 54.9 | 52.9 |
| Handpump/well | 36.4 | 36.1 ^††^ |
| Toilet facility |  |  |
| Own flush toilet | 04.0 | 04.8 |
| Own pit toilet | 04.2 | 05.7 |
| Shared toilet | 01.1 | 01.0 |
| No toilet | 90.7 | 88.6 ^††^ |

NS = non-significant difference between member and non-member sub-cohorts

*** Significance of difference between member and non-member sub-cohorts p<0.1 (ANOVA)

*** Significance of difference between member and non-member sub-cohorts p<0.05 (ANOVA)

*** Significance of difference between member and non-member sub-cohorts p<0.001 (ANOVA)

^†††^ Significance of difference in distribution between member and non-member sub-cohorts p<0.05 (Pearson Chi-square)

^†††^ Significance of difference in distribution between member and non-member sub-cohorts p<0.001 (Pearson Chi-square)

^a^ Comparison of the two sub-cohorts in our dataset: households where at least one person in the household was member of a Self-Help Group (SHG) linked to one of the related NGOs (member sub-cohort) and households where no one in the household was member of a Self-Help Group (SHG) linked to one of the related NGOs (non-member sub-cohort).

^b^ SE = Standard Error.

^c^ Income is proxied as monthly per capita consumer expenditure through questions on many items of household expenditure and expressed in Purchasing Power Parity International Dollar.

^d^ Asset-index is a proxy for socioeconomic status based on various aspects of household assets. The index is calculated using a principal component analysis (PCA).

^e^ Caste is a proxy for socioeconomic status in India. Scheduled Castes (Dalits and those sometimes labelled “untouchable”) are considered at the bottom of caste hierarchy. The list of Other Backward Castes is quite dynamic and changes from time to time in many states. All other castes are described here as General Caste.
