# Supplementary material for: Hardship financing of healthcare among rural poor in Orissa, India
Source: BMC Health Serv Res. 2012 Jan 27;12:23. doi: 10.1186/1472-6963-12-23 (PMC3317855; doi:10.1186/1472-6963-12-23)
Supplement: Additional file 2 — Morbidity, healthcare availability, utilization and cost disaggregated for members and non-members. This file contains the same information of Table 2 on morbidity, healthcare availability, utilization and cost but separate for the member and non-member sub-cohorts (as defined in the methods section). [file 1472-6963-12-23-S2.DOC]

Additional File 2

Morbidity, healthcare availability, utilization and cost disaggregated for members and non-members

|  | Non-member  sub-cohort^a^ | Member  sub-cohort^a^ | |
| --- | --- | --- | --- |
|  | Mean (±SE)^b^ | Mean (±SE)^b^ | |
| Total health expenditure last year for household (PPP$)^c^ | 159.32 (±7.62)*^NS^ | 175.33 (±6.89)*^NS^ | |
| Distance to preferred hospital (in minutes) | 51.93 (±0.76)*^NS^ | 53.03 (±0.76)*^NS^ | |
| Distance to preferred primary care practitioner (in minutes) | 30.37 (±0.56)*^NS^ | 30.43 (±0.56)*^NS^ | |
|  |  |  | |
|  | % of total | % of total | |
| Household with chronic ill person | 09.7 | 11.3 ^††^ | |
| Household with hospitalization costs last year | 22.3 | 24.7 ^††^ | |
| Household with outpatient care costs last year | 82.9 | 84.7 ^††^ | |
| Household with maternity costs last year | 15.3 | 13.5 ^††^ | |
| Household with any healthcare costs last year | 84.2 | 85.9 ^††^ | |
| Hospital household usually goes to |  | |  |
| Private | 06.5 | 06.0 ^††^ | |
| Public | 93.5 | 94.0 ^NS^ | |
| Preferred primary care practitioner household usually goes to |  | |  |
| Traditional healer | 35.6 | 37.5 | |
| Government facility | 51.4 | 49.5 | |
| Unqualified private doctor (non-MBBS)^d^ | 07.8 | 07.4 | |
| AYUSH practitioner^e^ | 03.4 | 03.0 | |
| Qualified private doctor/specialist (MBBS) | 01.7 | 02.7 ^††^ | |

NS = non-significant difference between member and non-member sub-cohorts

^†††^ Significance of difference in distribution between member and non-member sub-cohorts p<0.1 (Pearson Chi-square)

^†††^ Significance of difference in distribution between member and non-member sub-cohorts p<0.05 (Pearson Chi-square)

^a^ Comparison of the two sub-cohorts in our dataset: households where at least one person in the household was member of a Self-Help Group (SHG) linked to one of the related NGOs (member sub-cohort) and households where no one in the household was member of a Self-Help Group (SHG) linked to one of the related NGOs (non-member sub-cohort).

^b^ SE = Standard Error.

^c^ Total health expenditure last year for household expressed in Purchasing Power Parity International Dollar.

^d^ Unqualified private doctor (non-MBBS) is a doctor practicing allopathic medicine without having a medical degree (Medical Bachelor and Bachelor of Surgery).

^e^ AYUSH is the aggregate of all qualified systems of traditional medicines in India: Ayurveda, Yoga and Naturopathy, Unani, Siddha and Homeopathy.
